# Effect of community‐initiated kangaroo mother care on breastfeeding performance in low birthweight infants: A randomized clinical trial

**DOI:** 10.1111/mcn.13419

**Published:** 2022-08-10

**Authors:** Bireshwar Sinha, Halvor Sommerfelt, Per Ashorn, Sarmila Mazumder, Sunita Taneja, Rajiv Bahl, Nita Bhandari

**Affiliations:** ^1^ Centre for Health Research and Development, Society for Applied Studies New Delhi India; ^2^ Center for Child, Adolescent and Maternal Health Research, Faculty of Medicine and Health Technology, Tampere University, and Tampere University Hospital Tampere Finland; ^3^ DBT/Wellcome India Alliance Clinical and Public Health Fellow Hyderabad India; ^4^ Department of Global Public Health and Primary Care, Centre for Intervention Science in Maternal and Child Health University of Bergen Bergen Norway; ^5^ Cluster for Global Health, Division for Health Services, Norwegian Institute of Public Health Oslo Norway; ^6^ Department of Maternal Newborn, Child and Adolescent Health World Health Organization Geneva Switzerland

**Keywords:** breastfeeding performance, infant, Kangaroo mother care, low birthweight

## Abstract

This individually randomized trial was conducted to estimate the effect of promoting community‐initiated kangaroo mother care (ciKMC) in low birthweight (LBW) infants on infant breastfeeding performance. It was designed as a substudy within a larger primary trial on ciKMC and infant survival. Five hundred fifty stable LBW mother‐infant dyads (1500−2250 g) who provided consent, were consecutively enroled for breastfeeding performance assessment. The ciKMC intervention included promotion and support of continuous skin‐to‐skin contact and exclusive breastfeeding (EBF) through home visits during the neonatal period. The primary outcome was effective breastfeeding performance indicated by an infant breastfeeding assessment tool score of ≥10 after the end of the neonatal period. As secondary outcomes, we reported maternal satisfaction related to infant breastfeeding, and EBF after the end of the neonatal period. We completed outcome assessments in 96% of participants. In the ciKMC arm, 92% of the infants showed effective breastfeeding performance against 81% in the control arm [adjusted prevalence ratio (aPR): 1.24, 95% confidence interval (CI): 1.16−1.32]. In the ciKMC arm, 65% of the mothers reported to be very satisfied with their infants' breastfeeding against 51% in the control arm (aPR: 1.22, 95% CI: 1.05−1.41). The proportion of infants practicing EBF was 89% in the ciKMC arm against 45% in the control arm (aPR: 1.62, 95% CI: 1.45−1.81). Our study findings suggest that promotion of ciKMC can improve effective breastfeeding, EBF and maternal satisfaction related to breastfeeding in LBW infants.

## Key messages

- In low birthweight (LBW) infants, breastfeeding can be challenging with low prevalence of exclusive breastfeeding (EBF).
- Our trial in North India among 550 LBW infants showed that promotion of community‐initiated Kangaroo mother care (ciKMC) can substantially improve infant breastfeeding performance.
- ciKMC promotion substantially improved EBF prevalence at the end of the neonatal period, number of breastfeeds per day and duration of each breastfeed. It also enhanced mother's satisfaction with their infant's breastfeeding performance.

## Graphical abstract

Our trial in North India among 550 low birthweight infants showed that promotion of Kangaroo mother care can substantially improve infant breastfeeding performance, exclusive breastfeeding and mother's satisfaction with their infant's breastfeeding performance.

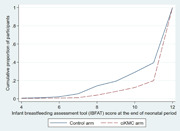

## 1 INTRODUCTION

The World Health Organization (WHO) recommends initiation of breastfeeding within 1 h of birth and exclusive breastfeeding (EBF) for the first 6 months of life for all infants including those born low birthweight (LBW) <2500 g (WHO, 2011, 2021). A meta‐analysis indicates that, compared to infants exclusively breastfed during the first 6 months of life, all‐cause mortality is 14 times higher in non‐breastfed infants and 5 times higher in partially breastfed infants (Sankar et al., 2015). In addition, breastfeeding is associated with reduced risk of breast cancer, ovarian cancer, and diabetes in the mothers (Victora et al., 2016). Mothers of infants born preterm, that is, before 37 completed weeks of gestation or LBW, often report perceived breastmilk insufficiency, making practice and continuation of EBF difficult (Edmond & Bahl, 2006; Maastrup et al., 2014; Mathur & Dhingra, 2009; Sethi et al., 2017). A multicountry cohort study reported that the practice of non‐EBF at 42 days of age was ~30% higher in Indian LBW infants than those with birthweight ≥2500 g (Patel et al., 2015). In a study in India among late preterm infants (34−36 weeks of gestation), EBF practice at the end of the first postnatal week after birth was reported to be 33%, with a feeling of insufficient milk as the most common reason for non‐EBF practice (Harsha & Kumar, 2017).

Achieving a high prevalence of EBF in the LBW or preterm infants can be challenging. Poor breastfeeding in these infants is associated with attachment or latching‐on difficulties, drowsiness, poor and intermittent sucking, poor coordination of sucking and swallowing and disorganized feeding behaviour (Dosani et al., 2016; Harsha & Kumar, 2017). Moreover, poor breastfeeding behaviour can also be associated with parental stress and perceived breastfeeding insufficiency in the mother (Dongre et al., 2010; Dosani et al., 2016). The WHO and the Government of India recommend Kangaroo mother care (KMC), an intervention encompassing skin‐to‐skin contact (SSC) and EBF, to improve survival in LBW babies (Conde‐Agudelo & Diaz‐Rossello, 2016; GOI, 2014; WHO, 2003). SSC following birth may promote early and improved attachment of the infant to the mother's breast, leading to successful breastfeeding (Dewey et al., 2003; Winberg, 2005). Our randomized controlled trial in India among 8402 LBW infants demonstrated that promotion of community‐initiated KMC (ciKMC) improves post‐enrolment neonatal survival by 30% (Mazumder et al., 2019). It is plausible that ciKMC promotion may improve breastfeeding performance in LBW infants, but high‐quality evidence from randomized trials is lacking.

Our primary objective was to test the hypothesis that promotion of ciKMC improves effective breastfeeding performance (Matthews, 1988) in LBW infants after the end of the neonatal period that is, 28 days after birth. As secondary objectives, we estimated the effect of promotion of ciKMC in LBW infants on the maternal perception of infant breastfeeding and the EBF prevalence after the end of the neonatal period.

## 2 METHODOLOGY

### 2.1 Study design and participants

**Figure 1:**
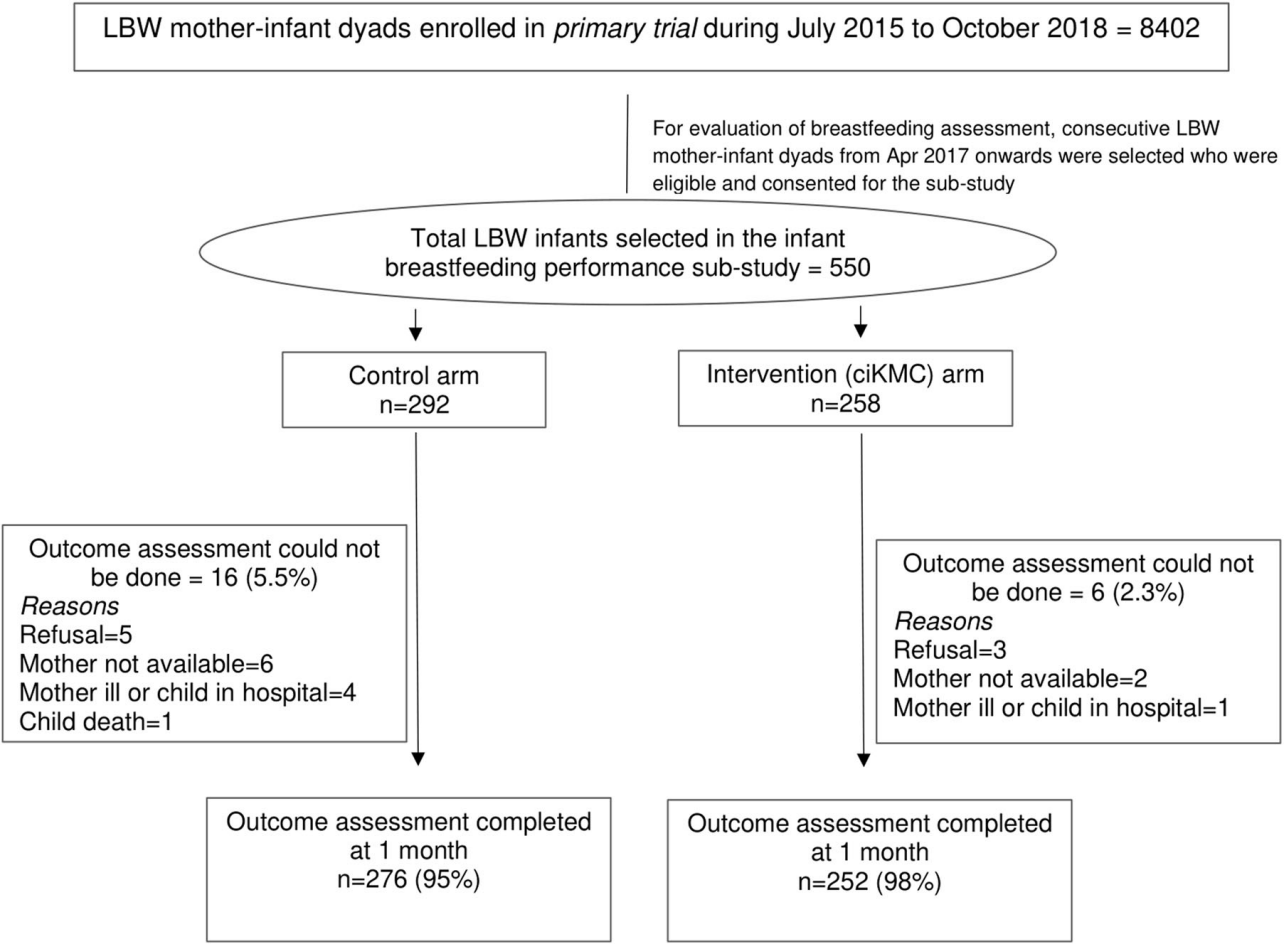
Participant flow in the trial. ciKMC, community‐initiated kangaroo mother care; LBW, low birthweight.

This individually randomized parallel arm trial was developed as a substudy within a larger primary trial (ClinicalTrials.gov NCT02653534) (Mazumder et al., 2017) specifically to assess infant breastfeeding performance. The primary trial was conducted in rural and semiurban low‐income populations of Faridabad and Palwal districts in Haryana, India. Newborns weighing 1500−2250 g and their mothers were screened for enrolment as early as possible, within 72 h of birth. Infants were excluded if KMC had already been initiated in a birth facility, not weighed within 72 h of birth, or they were unable to feed, had breathing problems, gross congenital malformations, reported less than normal movements (WHO, 2014) or mothers were not living with their babies or intending to move away over the next 6 months. Additionally, in this substudy, we excluded twins, triplets and infants in whom breastfeeding was not initiated. In the primary trial enrolments were done between July 2015 and October 2018. For infant breastfeeding performance assessment, we enroled consecutive eligible and consenting mother‐infant dyads from April 2017 onwards (Figure 1) in the substudy using the randomization sequence of the primary trial.

The randomization list was prepared by an off‐site statistician using random permuted blocks of variable size. Allocation of participant identification number was conducted by an independent coordinator using serially numbered opaque sealed envelopes (Mazumder et al., 2017, 2019).

### 2.2 Intervention and usual care

The ciKMC intervention comprised of promotion and support of continuous and prolonged SSC and EBF. The intervention delivery team visited homes of the infant‐mother dyads allocated to the ciKMC arm to initiate and support KMC on Days 1, 2, 3, 5, 7, 10, 14, 21 and 28 after birth to observe and solve any problems related to SSC or breastfeeding. Mothers were counselled to practice SSC for as long as possible during day and night, with the assistance of other family members. Visits continued till 28 days of age or until the baby wriggled out of the KMC position and no longer accepted SSC, if earlier. Referral of ill infants in both ciKMC and control arms was facilitated through government Accredited Social Health Activists (ASHAs) (NHM, 2014). All infants in both the ciKMC and control arms received home‐based postnatal care visits by ASHAs as implemented through the health system (MOHFW, 2014). Further details of the intervention delivery have been published previously (Mazumder et al., 2019).

### 2.3 Study outcomes and its assessment

Our primary outcome for the substudy was effective breastfeeding performance after the end of the neonatal period, indicated by a score of ≥10 in the infant breastfeeding assessment tool (IBFAT) (Khadivzadeh & Karimi, 2009; Matthews, 1988; Moore & Anderson, 2007). In addition, we report median IBFAT score, and the proportion of infants showing the score's individual components, that is, effective readiness to feed, rooting, fixing and sucking pattern. Each of these components are scored from 0 to 3 on a Likert scale, ‘0’ indicating the worst and ‘3’ indicating effective performance, with the total score ranging from 0 to 12 (Supporting Information: Appendix 1). IBFAT was developed by Matthews M. K. in 1988 (Matthews, 1988) has been found to be a reliable and validated tool for assessment of infant breastfeeding performance with high correlation (*R* > 0.7) with other available instruments like LATCH and the Mother Baby Assessment tool (Altuntas et al., 2014). It has been used in several previous trials (Moore & Anderson, 2007; Srivastava et al., 2014).

As secondary outcomes we assessed maternal satisfaction related to infant breastfeeding (based on 7 day recall) and EBF (24 h recall) at the end of the neonatal period (WHO & UNICEF, 2021). For assessment of maternal satisfaction related to infant breastfeeding, the mother was asked about her perception related to breastfeeding of the infant in the last 7 days if she was ‘very satisfied’, ‘satisfied’, ‘somewhat satisfied’ or ‘not satisfied’ with the way the baby fed during each feeding. This four‐point Likert scale was originally used by Matthews (1988) and thereafter adapted and has been used previously in different settings including in India (Matthews, 1988; Srivastava et al., 2014).

Outcome assessment specific to the substudy was conducted by a trained team of four workers who had at least graduate‐level education, hereby referred as ‘substudy outcome assessment team’. This team was not involved in intervention delivery or ascertainment of any other outcomes, including the duration of SSC, which was done by the primary trial outcome assessment workers. Training of the substudy outcome assessment team in breastfeeding assessment was conducted by a trainer certified in infant and young feeding counselling by the Breastfeeding Promotion Network of India. After training, a standardization exercise was done in which the IBFAT questionnaire scores assigned by the substudy outcome assessment team workers were matched with those of the certified trainer. An interclass correlation coefficient of >0.75 (Koo & Li, 2016) between the workers and the trainer for all the four components of the tool was a criterion to allow the workers to conduct the IBFAT assessment. All outcomes were assessed in the homes of the participants.

### 2.4 Statistical analysis

The sample size for the substudy was calculated prior to its initiation. With the assumption that 50% of the infants in the control arm would show effective breastfeeding (Kishore et al., 2009) and to be able to detect a minimum of 25% relative improvement in the in this proportion with 80% power, 95% confidence interval (CI) and 10% attrition, we enroled a total of 550 participants.

Analyses were conducted on an intent‐to‐treat basis using STATA version 16 (Stata Corporation). As outcome data were available in >95% of the study participants, we did not impute for missing data (Jakobsen et al., 2017).

We estimated prevalence ratios (PRs) for effective breastfeeding performance between the study arms using generalized linear models (GLM) of the binomial family with a log‐link. In addition, we estimated absolute risk difference and the number needed to treat. Maternal age, birth order, place of delivery, sex of the baby, weight at enrolment and preterm birth were considered potential confounding factors based on prior knowledge if associated with the primary outcome at *p* < 0.1 in univariable analysis. To estimate the adjusted prevalence ratio (aPR), we included a potential confounding factor if it were unequally distributed between the study arms at baseline (a priori defined as a relative difference of >10%) and the baseline IBFAT scores in the multivariable GLM analysis. Design effects of infants enroled from a single household were accounted for using Stata's robust variance estimator (cluster) option. We used the same analytical approaches for the other categorical study outcomes that is, maternal satisfaction related to infant breastfeeding (very satisfied), and EBF.

We conducted subgroup analyses, decided a priori, to estimate whether the effect of ciKMC on effective breastfeeding performance was different in preterm infants (<37 weeks gestation) compared with full‐term infants (≥37 weeks gestation). To quantify any biological interaction between ciKMC promotion and preterm birth, we estimated the absolute excess risk due to interaction using interaction term in the GLM analysis. Gestational age was estimated from the ultrasonography reports, when available, or based on the last menstrual period as documented in hospital records or as per maternal recall, in the given order of preference.

For IBFAT scores, we reported the median and interquartile range, given its asymmetric left‐skewed distribution. We used the Wilcoxon rank‐sum test to evaluate the hypothesis that promotion of ciKMC was associated with higher IBFAT scores. To explore the consistency of our analysis across different cut‐offs of IBFAT score, we plotted the cumulative density frequency of the total IBFAT score after the end of the neonatal period across study arms.

## 3 RESULTS

The baseline characteristics were similar in the two study arms other than for home delivery, and birth order (≥5), where the relative differences in proportions between study arms exceeded 10% (Table 1). Among the 550 enroled participants, we completed outcome assessments in 98% (252/258) of the infants in the ciKMC arm and 95% (276/292) of the infants in the control arm (Table 2).

**Table 1:** Baseline characteristics of participants in the control and intervention arm^a^

	Control (*N* = 276)	ciKMC (*N* = 252)
Variables	*n* (%)	*n* (%)
Household characteristics		
Wealth quintiles		
Least poor	53 (19.2)	60 (23.8)
Less poor	61 (22.1)	50 (19.8)
Poor	53 (19.2)	49 (19.4)
Very poor	58 (21.0)	52 (20.6)
Most poor	51 (18.5)	41 (16.3)
Religion: Hindu	225 (81.8)	204 (80.9)
Median number of family members (IQR)	7 (5−9)	7 (5−10)
Maternal and paternal characteristics		
Mean (SD) maternal age in years	23.4 (3.7)	23.2 (3.3)
Mean (SD) paternal age in years	26.9 (4.6)	26.4 (4.6)
Maternal education: Median (IQR) years of schooling	7 (0−10)	7 (0−10)
Birth related characteristics		
Home delivery	47 (17.0)	36 (14.3)
Birth order		
1	96 (34.9)	96 (38.1)
2−4	151 (54.9)	136 (54.0)
≥5	28 (10.2)	20 (7.9)
Infant characteristics		
Sex of the baby: Female	147 (53.3)	141 (55.9)
Mean (SD) weight at enrolment in gram	2076 (166)	2095 (154)
Mean (SD) gestational age in weeks^b^	35.6 (2.2)	35.7 (2.0)
Proportion born preterm (<37 weeks)	178 (64.9)	164 (65.1)

Abbreviation: ciKMC, community‐initiated kangaroo mother care.

^a^ 65% (360/550) had an ultrasound for gestational age assessment.

^b^ Data presented are number/percentages unless indicated otherwise.

**Table 2:** Effect of community‐initiated kangaroo mother care on effective infant breastfeeding at the end of neonatal period

Outcome variable	Population	Control	ciKMC	Unadjusted PR	Adjusted^a^ PR
		*n*/*N* (%)	*n/N* (%)	(95% CI)	(95% CI)
Effective infant breastfeeding (IBFAT score ≥10)	All infants	223/276 (80.8)	232/252 (92.1)	1.14 (1.06−1.22)	1.24 (1.16−1.32)
	Preterm infants	136/178 (76.4)	147/164 (89.6)	1.17 (1.06−1.29)	1.30 (1.18−1.42)
	Term infants	87/98 (88.8)	85/88 (96.6)	1.08 (1.00−1.18)	1.13 (1.07−1.19)

Abbreviations: CI, confidence interval; ciKMC, community‐initiated kangaroo mother care; IBFAT, infant breastfeeding assessment tool; IQR, interquartile range; PR, prevalence ratio.

^a^ Adjusted for place of delivery, birth order and baseline IBFAT score. The design effect of more than one infant being included from a single household was accounted for by using Stata's cluster option to obtain a robust variance estimator.

All mothers in the ciKMC arm practiced SSC, while 8.7% (24/276) of the mothers in the control arm reported SSC practice. In the ciKMC arm, the median (interquartile range [IQR]) age of the infant at ciKMC initiation was 48 (19−72 h); the mean (SD) duration of SSC practice was 28 (2.4) days with 12 (3.8) hours per day. In the control arm, the mean (SD) duration of SSC practice was 2 (7.1) days with 1 (2.1) hour per day.

The median (IQR) IBFAT score at enrolment was 11 (9−12) both in the ciKMC arm and in the control arm infants. At the end of the neonatal period (mean age 28 days in both ciKMC and control arms), 92% (232/252) of the infants in the ciKMC and 81% (223/276) in the control arm showed effective breastfeeding performance. The aPR (95% CI) for effective breastfeeding performance adjusted for potential confounding baseline factors (place of delivery and birth order) and baseline IBFAT score was 1.24 (1.16−1.32), corresponding to an effect of 24% (16%−32%); the unadjusted PR was 1.14 (1.06−1.22). The absolute unadjusted risk difference (95% CI) for effective breastfeeding performance was 11.2% (5.5%−16.9%) corresponding to a number needed to treat of 9 infants (6−18).

**Figure 2:**
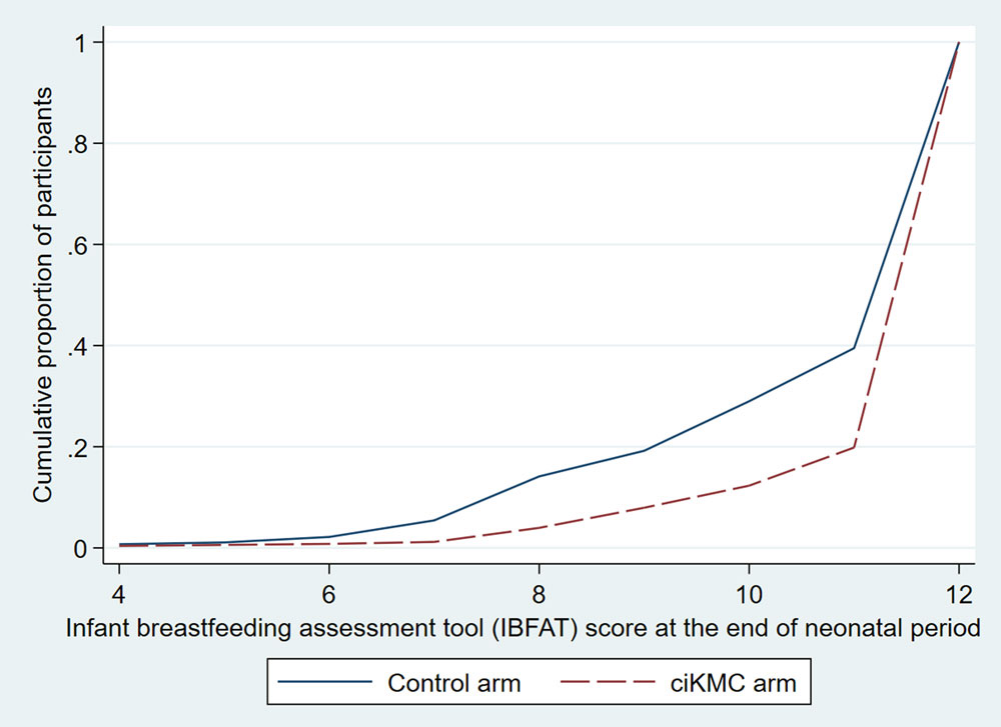
Cumulative frequency plot showing IBFAT^1^ scores at the end of neonatal period. ciKMC, community‐initiated kangaroo mother care; IBFAT, infant breastfeeding assessment tool.

At the end of the neonatal period, the median (IQR) of IBFAT score was 12 (12−12) in the ciKMC arm and 12 (10−12) in the control arm infants (*p* < 0.001). The cumulative frequency plot showed a right shift of the IBFAT score in the ciKMC arm compared with the control arm (Figure 2), showing that at any given cut‐off of the IBFAT score, a higher proportion of infants in the ciKMC arm was above the cut‐off score (Supporting Information: Table 1).

A higher proportion of infants in the ciKMC arm than the control arm showed effective readiness to feed (88% vs. 80%; aPR: 1.13, 95% CI: 1.06−1.21), effective rooting (91% vs. 83%; aPR: 1.18, 95% CI: 1.12−1.25), effective fixing (91% vs. 73%; aPR: 1.30, 95% CI 1.21−1.40) and effective sucking pattern (87% vs. 67%; aPR: 1.32, 95% CI: 1.21−1.45) (Supporting Information: Table 2).

In the subgroup of babies born preterm, the aPR (95% CI) of effective breastfeeding performance in the ciKMC arm was 1.30 (1.18−1.42) versus 1.13 (1.07−1.19) among full‐term infants. Absolute excess risk due to interaction between preterm birth and ciKMC for the primary outcome was estimated to be 0.05 (95% CI: −0.05 to 0.16).

In the ciKMC arm, 65% (164/252) of the mothers reported to be very satisfied with their infant's breastfeeding at the end of the neonatal period. In the control arm, the corresponding proportion was 51% (141/276, aPR: 1.22, 95% CI: 1.05−1.41, Table 3). Breast or nipple problems at the end of the neonatal period was reported by 5.1% (13/252) of the mothers in the ciKMC arm and 8.7% (24/276) of the mothers in the control arm (PR: 0.58, 95% CI: 0.30−1.11).

**Table 3:** Effect of community‐initiated kangaroo mother care on mother's reported perception on infant breastfeeding and breastfeeding prevalence (24 h recall) at the end of neonatal period

Outcomes	Control *N* = 276 *n* (%)	ciKMC *N* = 252 *n* (%)	Unadjusted PR (95% CI)	Adjusted PR (95% CI)
Mother's reported perception on infant breastfeeding (7‐day recall)				
Very satisfied	141 (51.1)	164 (65.1)	1.28 (1.10−1.48)	1.22 (1.05−1.41)^a^
Satisfied	110 (39.8)	83 (32.9)	Reference^b^	Reference^b^
Somewhat satisfied	16 (5.8)	4 (1.6)		
Not satisfied	9 (3.3)	1 (0.4)		
Breastfeeding rates (24 h recall)				
Exclusive	123 (44.6)	225 (89.3)	1.61 (1.43−1.80)	1.62 (1.45−1.81)^c^
Predominant	81 (29.4)	22 (8.7)	Reference^d^	Reference^d^
Partial	42 (15.2)	5 (1.9)		
No	0 (0.0)	0 (0.0)		

Abbreviations: CI, confidence interval; ciKMC, community‐initiated kangaroo mother care; PR, prevalence ratio.

^a^ Adjusted for place of delivery and birth order and mother's reported perception on breastfeeding at baseline.

^b^ For calculation of the prevalence ratio for ‘very satisfied’, all other categories are clubbed as the reference category.

^c^ Adjusted for place of delivery and birth order.

^d^ For calculation of the prevalence ratio for exclusive breastfeeding, all other categories are considered as nonexclusive breastfeeding.

The mean interval between birth and breastfeeding initiation was 4.4 (9.8) h in the ciKMC arm infants and 4.7 (10.5) h in the control arm infants. The proportion of infants practicing EBF at the end of the neonatal period was 89% (225/252) in the ciKMC arm and 45% (123/276) in the control arm (aPR: 1.62, 95% CI: 1.45−1.81, Table 3). The reported median (IQR) number of breastfeeds per day (24 h recall) was 12 (12−14) in the ciKMC arm and 11 (9−14) in the control arm (*p* < 0.001). The reported mean (SD) duration of each breastfeed was 15.5 (5.1) min in the ciKMC arm and 10.1 (5.1) min in the control arm infants (mean difference: 5.4, 95% CI: 4.5−6.3).

## 4 DISCUSSION

We aimed to estimate the effect of ciKMC promotion among LBW infants on effective breastfeeding performance, maternal satisfaction related to infant breastfeeding and EBF at the end of the neonatal period. In a sample of 550 stable LBW infants included in our trial, the prevalence of effective breastfeeding performance was substantially higher in the ciKMC arm than in the control arm. Our findings indicate that promoting ciKMC for 9 LBW infants would result in 1 more infant breastfeeding effectively. The ciKMC infants showed improved performance in all four components of the IBFAT, that is, readiness to feed, rooting, fixing and sucking pattern. ciKMC promotion also enhanced the mothers' satisfaction with their infant's breastfeeding performance. EBF at the end of the neonatal period, reported number of breastfeeds per day, and the duration of each breastfeed was substantially higher among ciKMC arm infants compared to among the control arm infants.

In our study, selection bias is unlikely, because of effective randomization, allocation concealment and low attrition. To minimize misclassification bias, we conducted a standardization exercise prior to the IBFAT assessment. Some of the outcome measurements were based on mothers recall like mother's perception of infant breastfeeding (7‐day recall) and EBF (24 h recall). The short recall periods minimize the possibility of recall bias. To minimize observer's bias, IBFAT assessment at the end of the intervention period was done by an independent trained team, unaware of trial arm allocation and not involved in intervention delivery. However, we cannot rule out that the unmasked nature of the trial might make it possible to guess the trial arm allocation with a resultant small observer bias in a few cases. Given the overall low risk of bias, we believe that our findings are internally valid. We therefore believe that the promotion of ciKMC, in low resource settings such as ours, can substantially improve breastfeeding performance, maternal satisfaction with breastfeeding and EBF prevalence in stable LBW infants.

Previous studies have used IBFAT to examine the effect of SSC practice in healthy or term infants in hospital settings on breastfeeding. A randomized trial in North India among 298 healthy mother‐infant dyads reported that IBFAT score at 6 weeks was increased in infants who were supported to practice SSC for at least 2 h. Likewise, a randomized trial in Nashville, Tennessee, among full‐term infants, reported a substantially higher IBFAT score in the SSC arm infants compared to controls (Moore & Anderson, 2007). Similarly, a meta‐analysis including three randomized trials conducted in India, Italy and the USA, reported higher IBFAT scores in healthy infants practicing SSC compared to those with routine care in hospital settings (Ghojazadeh et al., 2019). Another study in Iran showed that full‐term infants randomized to practice SSC for 1 h after birth had higher effective readiness to feed, effective sucking, effective latching and effective rooting (Beiranvand et al., 2014). Our trial findings substantiate the observations from such studies and provide rigorous evidence that promotion of community initiated KMC in the population of stable LBW infants can substantially improve breastfeeding performance.

Studies have also reported the effect of SSC or KMC on infant breastfeeding rates and maternal satisfaction. A previous meta‐analysis reported that practice of SSC or KMC initiated in hospitals in stable low birthweight infants is associated with higher EBF rates during the first 6 months of life and longer duration of breastfeeding (Conde‐Agudelo & Diaz‐Rossello, 2016; Moore et al., 2016). An earlier study in North India reported that the scores of mothers' perception about their infant's breastfeeding, as measured on a four‐point Likert scale, were substantially higher in the SSC arm compared to controls (Srivastava et al., 2014). Our findings concur with these findings and suggest that KMC initiated in the community or can improve EBF prevalence and maternal satisfaction with breastfeeding.

Biologically, the concept of SSC evolved from animal studies which suggested that maintenance of the maternal milieu after birth of small or premature babies may help to promote innate behaviours in the newborn and the mother, leading to successful breastfeeding and increased survival. Closeness with the mother is associated with regulation of different aspects of neonatal physiology, including behavioural, cardiorespiratory, digestive and hormonal systems (Hofer, 2006; Moore et al., 2016). Small‐born infants, that is, those who are LBW or preterm, are often separated from their mother after birth, which may affect development of these physiological and behavioural systems. The effect of ciKMC on effective infant breastfeeding as well as improved maternal satisfaction is plausible because of improved feedback mechanisms between the mother and the baby, better mother‐infant bonding, reduced stress and improved breastfeeding confidence (Lau, 2018). The lower proportion of reported breast or nipple related problems in ciKMC arm mothers may indicate a maternal benefit, although our estimate for that effect was statistically imprecise. The observed tendency towards a higher PR of effective breastfeeding in preterm than in term infants may be relevant given that the former group of infants are at a higher risk of non‐EBF (Ayton et al., 2012). Nonetheless, we acknowledge the limitations of this subgroup analysis in that we had not stratified the randomization based on preterm or term births and the statistical precision of the absolute excess risk due to interaction was low.

Despite the low likelihood of bias and error in our trial, there were some limitations. Our study was limited to the population of stable LBW infants weighing 1500−2250 g in a low to middle income setting in India. The findings may not be generalizable to unstable or very low birthweight infants <1500 g or in different settings. For assessment of infant breastfeeding performance, we used the IBFAT which has been widely used for this purpose but is not a standard acceptable tool in all settings. Observation‐based assessment of infant breastfeeding is associated with some degree of subjectivity and therefore estimation of volume of breast milk intake with newer methods like stable‐isotope technology may add value. For assessment of breastfeeding performance, several other tools are available (Altuntas et al., 2014; Ingram et al., 2015), and the development of a standard tool to assess infant breastfeeding performance to allow comparisons across studies and settings will be important in future.

## 5 CONCLUSION

Our study findings support the promotion of ciKMC as an intervention for LBW babies to improve effective breastfeeding, EBF and maternal satisfaction related to breastfeeding. Given the benefits, integration of ciKMC within the essential newborn care programs in low‐middle income settings should be encouraged.

## AUTHOR CONTRIBUTIONS

Bireshwar Sinha and Halvor Sommerfelt had full access to all the data in the study and take responsibility for the integrity of the data and the accuracy of the data analysis. *Concept and design*: Bireshwar Sinha and Halvor Sommerfelt. *Acquisition, analysis or interpretation of data*: Bireshwar Sinha, Halvor Sommerfelt, Per Ashorn, Sarmila Mazumder and Sunita Taneja. *Drafting of the manuscript*: Bireshwar Sinha, Halvor Sommerfelt and Per Ashorn. *Critical revision of the manuscript for important intellectual content*: All authors. *Statistical analysis*: Bireshwar Sinha, Halvor Sommerfelt and Per Ashorn. *Obtained funding*: Bireshwar Sinha, Nita Bhandari and Halvor Sommerfelt. *Supervision*: Halvor Sommerfelt, Ashorn, Per Bahl and Nita Bhandari.

## CONFLICT OF INTEREST

The authors declare no conflict of interest.

## ETHICS STATEMENT

Ethics approval was obtained from the Institutional Ethics Review Committee and the Regional Committee for Medical and Health Research Ethics (REK) in Western Norway. The substudy was separately registered with Clinical trials registry‐India (CTRI/2017/04/008430). Written informed consent was obtained from the mothers of the eligible infants before enrolment. The participants were identified by study numbers to assure confidentiality and anonymity. The study is reported as per the Consolidated Standards of Reporting Trials (CONSORT) reporting guideline (Appendix 2) (Schulz et al., 2010).

## Supporting information

Supporting information.

## Data Availability

The primary custodian of the data is Centre for Health Research and Development, Society for Applied Studies (CHRD SAS), India. As per the Institutional policy deidentified data will be made available on request for the purpose of checking consistency or supporting the analyses presented in this scientific manuscript.
